# Synthesis and Performance Testing of Maleic Anhydride–Ene Monomers Multicomponent Co-Polymers as Pour Point Depressant for Crude Oil

**DOI:** 10.3390/polym15193898

**Published:** 2023-09-27

**Authors:** Dong Yuan, Qingfeng Liu, Wenhui Zhang, Ran Liu, Chenxi Jiang, Hengyu Chen, Jingen Yan, Yongtao Gu, Bingchuan Yang

**Affiliations:** 1 College of Chemistry and Chemical Engineering, Qilu Normal University, Jinan 250013, China; liuranbjt@163.com; 2 Shengli Oil Field Zhongsheng Petroleum Development Company, Dongying 257237, China; wolfmanliu007@163.com; 3 School of Chemistry and Chemical Engineering, Liaocheng University, Liaocheng 252000, China; zwh2963105101@163.com (W.Z.); jcx20010331@163.com (C.J.); qicechenyu@163.com (H.C.); 4 Gudong Oil Production Plant, Shengli Oilfield Company, SINOPEC, Dongying 257237, China; yanjingen@163.com (J.Y.); guyongtao@163.com (Y.G.)

**Keywords:** crude oil, pour point depressant, maleic anhydride, ene monomers, pour point depressant mechanism

## Abstract

To address the issue of pipeline blockage caused by the formation of waxy deposits inside pipelines, hindering the flow of petroleum in the Shengli oilfield, eight new-style polyacrylic acid pour point depressants (PPD) for Shengli crude oil were prepared by maleic anhydride and ene monomers with different polar and aromatic pendant chains. The synthesized Pour Point Depressants were characterized by Fourier transform infrared spectroscopy (FTIR), nuclear magnetic resonance (NMR), gel permeation chromatography (GPC), and polarizing optical microscopy (POM). The results were promising and demonstrated that any type of pour point depressant exhibited excellent performance on high-pour-point crude oil. The reduction in pour-point after additive addition was largely dependent on the polymer structure. Notably, polymers containing long alkyl side chains and aromatic units displayed the most impressive performance, capable of depressing the pour point by 12 °C.

## 1. Introduction

The Shengli oilfield is located in the northeast region of China, which is a typical cold climate zone with the lowest temperature dropping to −10 °C, and the average winter temperature ranging between 2 and −3 °C [1,2,3]. Particularly during winter, the extremely low temperatures facilitate the crystallization of wax present in crude oil, leading to the formation of waxy substances that precipitate within the pipeline system and consequently elevate the viscosity of the crude oil [4,5,6,7]. According to surveys [8,9], the wax content in Shengli crude oil is relatively high, generally around 10%, and in some areas, it can even reach more than 25%. This makes it extremely difficult to transport crude oil at low temperatures. China is one of the world’s largest oil-consuming countries [10], and so, it is urgent to solve the problem of low-temperature transportation of crude oil.

The pour point is the temperature at which crude oil becomes viscous and no longer flows under certain conditions [11]. Above the pour point, crude oil can still flow, while below the pour point, crude oil becomes viscous and difficult to flow [12]. In a low-temperature environment, the pour point of crude oil increases, causing it to solidify and crystallize, making it difficult to flow [13]. Therefore, the pour point is an important indicator of crude oil that needs to be tested and controlled to address the challenges associated with the low-temperature transportation of crude oil [14].

The fundamental way to solve this problem is to add pour point depressants to reduce the pour point of crude oil [15,16,17,18]. Crude oil pour point depressants are usually high molecular weight polymers containing a large number of polar functional groups such as hydroxyl and carboxyl groups [19,20]. By means of van der Waals forces, particularly hydrophobic interactions, the polymer interacts with the hydrocarbons in crude oil, forming distinct associations, in which the hydrophilic moieties are oriented on the outside, preventing the agglutination and subsequent precipitation of the waxy molecules [21,22]. There are three mature viewpoints on the principle of crude oil pour point depressants: crystal nucleation theory, adsorption theory, and eutectic theory [23,24,25,26]. These theories effectively explain the role of crude oil pour point depressants in improving the flowability of crude oil, laying a foundation for the subsequent preparation and development of pour point depressants [27,28,29].

Recently, researchers have been gradually exploring the connection between the structure and mechanism of pour point depressants, leading to the development of a wider range of pour point depressants [30,31,32]. These include (poly)methacrylate-based pour point depressants with improved shear resistance and a variety of maleic anhydride-based pour point depressants with diverse functionalities. Wu et al. synthesized four PPDs with maleic anhydride and its derivatives of different polar and aromatic pendant chains, which are binary copolymers [33]. Fang et al. prepared the ternary copolymers by mixing the aminated copolymer and the composite commercial ethylene-vinyl acetate copolymers (EVA) [34]. Based on previous work, we synthesized eight new-style pour point depressants by polymerizing maleic anhydride and various ene monomers, including acrylic acid and acrylic ester. We tested the effectiveness of these pour point depressants using Shengli crude oil with a pour point of 32 °C and the results demonstrated that any type of pour point depressant exhibited excellent performance on high-pour-point crude oil. Additionally, we explored the mechanism of action of the PPDs and discussed the impact of different functional groups and concentrations on their performance.

## 2. Materials and Methods

### 2.1. Materials

Acrylic acid, methyl acrylate, propyl acrylate, 2-butene-1-methyl acrylate, maleic anhydride, methyl methacrylate, vinyl acetate, octadecyl acrylate, nonadecyl acrylate, benzyl alcohol, anhydrous ethanol, benzyl alcohol, diethyl maleate, dibenzyl maleate, sulfuric acid, N,N-dimethylformamide (DMF), benzoyl peroxide (BPO), and petroleum ether were purchased from Shanghai in China. These are analytical reagents and were used as received without further purification.

Shengli (SL) waxy crude oil was selected to evaluate the efficiency of polymeric additives [35]. The physicochemical characteristics of SL crude oil are listed in Table 1.

### 2.2. Preparation of the Eight Pour Point Depressant (PPD)

**Figure 1:**
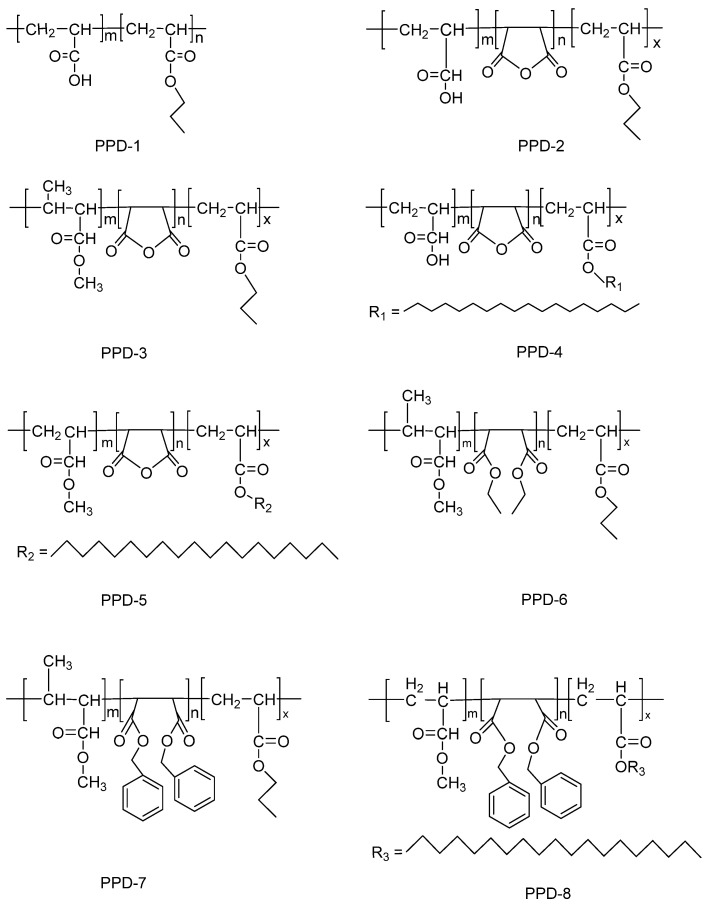
Chemical structures of the prepared pour-point depressants: PPD-1, PPD-2, PPD-3, PPD-4, PPD-5, PPD-6, PPD-7, and PPD-8.

Maleic anhydride can easily copolymerize with alkene monomers and react with alkyl alcohols and amines, making it possible to prepare pour point depressants by copolymerizing maleic anhydride with different monomers. We polymerized maleic anhydride with different ene monomers such as acrylic acid and acrylic ester, and added various polar and aromatic units into the polymer backbone to prepare eight copolymers with good performance (Figure 1).

#### 2.2.1. Preparation of PPD-1

The acrylic acid and propyl acrylate (1:1, molar ratio, Table 2) were dissolved in 500 mL of solvent DMF. A mixture of 0.8 g of initiator BPO and 20 mL of petroleum ether was prepared and slowly added to the reaction vessel using a dropping funnel. The reaction was carried out under constant temperature at 80 °C for 2 h. After the polymerization reaction was complete, impurities were removed using a rotary evaporator, and the product PPD-1 was obtained after cooling to room temperature.

#### 2.2.2. Preparation of PPD-2

The acrylic acid, the propyl acrylate, and the maleic anhydride (3:3:1, molar ratio, Table 2) were added to a three-neck flask. Solvent DMF was then poured into the flask. During the reaction, a mixture of petroleum ether and initiator BPO was added dropwise using a dropping funnel (the amount of initiator BPO was 0.1% of the total monomer mass). The reaction was maintained at a constant temperature of 80 °C for 2 h, and the product was obtained by rotary evaporation.

#### 2.2.3. Preparation of PPD-3, PPD-4, PPD-5

The synthetic methods of PPD-3, PPD-4, and PPD-5 are similar to the preparation method of PPD-2.

#### 2.2.4. Preparation of PPD-6

Diethyl succinate was prepared by directly reacting 1 mol of maleic anhydride with 2 mol of anhydrous ethanol in the presence of sulfuric acid (1%) as a catalyst heating at 75 °C. After the reaction was complete, the catalyst and unreacted materials were removed by washing with distilled water, and the pure ester was obtained. The methyl methacrylate, the propyl acrylate, and the pure ester prepared (3:3:1, molar ratio, Table 2) were added to a three-neck flask. Solvent DMF was then poured into the flask. During the reaction, a mixture of petroleum ether and initiator BPO was added dropwise using a dropping funnel (the amount of initiator BPO was 0.1% of the total monomer mass). The reaction was maintained at a constant temperature of 80 °C for 2 h, and the product was obtained by rotary evaporation.

#### 2.2.5. Preparation of PPD-7

Dibenzyl maleate was prepared by directly reacting 1 mol of maleic anhydride with 2 mol of benzyl alcohol in the presence of sulfuric acid (1%) as a catalyst heating at 75 °C. After the reaction was complete, the catalyst and unreacted materials were removed by washing with distilled water, and the pure ester was obtained. The remaining steps were similar to the preparation method of PPD-6.

#### 2.2.6. Preparation of PPD-8

Based on the synthesis of the binary copolymerized pour point depressant PPD-1, ternary copolymerized pour point depressants were obtained by inserting a third monomer, which improved the structure of PPD and had better performance. Compared with PPD-1, the structure of PPD-2 inserted maleic anhydride monomer; meanwhile, PPD-3 can be obtained by adding a methyl group in the structure of acrylic acid monomer. A higher pour point depression effect of the polymeric improver (PPD-4, PPD-5) can be obtained by exerting a long alkyl side chain on acrylic ester monomer. PPD-6 can be produced by ring-opening maleic anhydride. Aromatic rings were incorporated into ring-opening maleic anhydride groups to attain PPD-7. Both insert aromatic side chains in maleic anhydride structure and add pendant chains on acrylate monomer can fabricate PPD-8.

Generally speaking, both the copolymers prepared by us and Wu et al. [21] possessed long-chain alkyl and maleic anhydride structures. In comparison to the pour point depressants obtained by Wu et al., our PPD-1 exhibited similarities, as both were binary copolymers. Additionally, the PPD-4 we synthesized and the POM produced by Wu et al. shared the monomeric structure of octadecyl acrylate and maleic anhydride. The presence of ring-opening maleic anhydride groups with aromatic rings and long alkyl side chains was observed in our PPD-8 and the POMB obtained by Wu et al. However, the difference lies in the fact that, apart from PPD-1, the copolymers we synthesized were ternary copolymers. We employed various ene-monomers co-polymerized with maleic anhydride, facilitating a more effective action of the pour point depressant. This was best illustrated by the outstanding performance of PPD-8 in reducing the pour point by 12 °C at a concentration of 100 ppm.

### 2.3. Characterization of the Products

Gel permeation chromatography (GPC) (Waters Model 1515) was used to estimate the weight-average molecular weight (Mw) of the produced PPD. Tetrahydrofuran (THF) was used as the mobile phase at a flow rate of 1 mL/min and a temperature of 30 °C. Polystyrene was used as the standard. The Fourier transform infrared spectrometry (FT-IR) analyses were measured on a Nicolet 5700 spectrophotometer (Nicolet, Thermo Fisher, Waltham, MA, USA) in the range 400–4000 cm^−1^. ^1^H NMR and ^13^C NMR spectra were recorded on a 400 MHz (Bruker, Germany) instrument, and CDCl_3_ (7.26 ppm for ^1^H NMR, 77.16 ppm for ^13^C NMR) was used as a reference (Supplementary Materials).

### 2.4. The Selection of Pour Point Depressants

Firstly, 400 mL of oil sample was taken in a conical flask and placed in a water bath at 60 °C to completely melt it, stirring evenly. Secondly, four dry and clean test tubes were taken and 10 g of crude oil was added to each tube. Three of the tubes added 0.01 g of pour point depressant as the experimental group (concentration of 100 ppm), while the remaining tube served as the control group without the PPD. They were stirred evenly and placed in a water bath at 60 °C for 2 h to allow the PPD to fully take effect. Lastly, according to the national standard GB/T3535-83 [36], the pour point test was performed on the oil samples using a KDND-801 pour point tester, the data were recorded, and the average decrease in pour point among the three test tubes was calculated as the final result. This experiment was conducted in parallel for eight sets of PPD products, identifying the PPD product with the most effective performance.

### 2.5. Test Concentration of Pour Point Depressants

To determine the optimal concentration of the selected PPD, the experimental procedure was as follows: Firstly, 100 mL of oil sample was added to a conical flask and placed in a water bath at 60 °C for complete melting, and it was stirred thoroughly. Then, six dry and clean test tubes were taken, and 10 g of crude oil was added to each tube. Five of the tubes were individually added with PPD at concentrations of 50 ppm, 100 ppm, 200 ppm, 300 ppm, and 500 ppm, respectively. The remaining tube served as a blank control without the addition of any PPD. After thorough stirring, the test tubes were placed in a water bath at 60 °C for 2 h to allow the PPD to fully take effect. Lastly, the pour point of the oil samples was tested using a KDND-801 pour point tester, and the data were recorded to determine the best concentration of the selected pour point depressant.

### 2.6. Microscopy Studies

Polarizing optical microscopy (POM) was used to examine the wax crystal structure of crude oil before and after the addition of the PPD at standard temperature, and images were captured using a computer.

## 3. Results and Discussion

### 3.1. Polymer Characterization

#### 3.1.1. GPC of PPD

The average molecular weight (M_w_) of the eight pour point depressants prepared was determined and the yields of these polymers are shown in Table 2. According to expert research, the molecular weight of the PPD should be within a certain range, typically between 4000 and 100,000 [30]. When the molecular weight of the PPD was below 2000, there was generally no significant effect on decreasing the pour point. Conversely, when the molecular weight exceeded 500,000, the effect was also poor for the performance activity mechanism. From Table 3, it can be observed that the average molecular weights of the PPD were all within the range of 34,958–42,339, which falls within the effective range of perfect performance. Within this range, the molecular weight was moderate, providing sufficient coverage and adhesion to effectively inhibit wax crystal growth and crystallization.

#### 3.1.2. IR Spectroscopy Analysis of PPD

**Figure 2:**
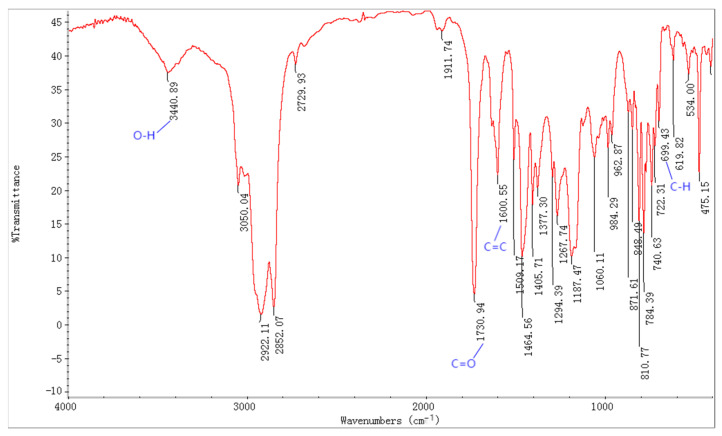
FT-IR spectra of PPD-8.

The chemical structure of the pour point depressants was determined by FT-IR spectroscopy. The FT-IR spectrum of PPD-8 is shown in Figure 2. The O-H absorption peak can be seen in 3440 cm^−1^. The absorption peak of aromatic ring C=C was 1600 cm^−1^. The carbonyl stretch vibration peak can be seen in 1730 cm^−1^. The characteristic C-H of aromatic ring absorption peak at 699 cm^−1^. It was confirmed that the acrylic ester and maleic anhydride had copolymerized and proved that we chose the right selected method to copolymerized. Additionally, the results obtained from other Fourier Transform Infrared Spectroscopy (FT-IR) analyses also demonstrated the successful execution of the synthesis process.

#### 3.1.3. Nuclear Magnetic Resonance Spectroscopy of PPD

In order to further confirm the structure of the pour point depressants, we also obtained nuclear magnetic resonance (NMR) spectra, including ^1^H NMR and ^13^C NMR.

^1^H NMR and ^13^C NMR Analysis:

PPD-1, pale yellow oil; ^1^H NMR: δ 3.9 (m, 1H), δ 2.2 (m, 1H), δ 1.6 (m, 2H), δ 1.2 (m, 27H), δ 0.8 (m, 2H); ^13^C NMR: δ 32.0, 29.8, 29.7, 29.4, 25.9, 22.7, 14.2.

PPD-2, pale yellow oil; ^1^H NMR: δ 7.4 (m, 1H), δ 7.1 (m, 1H), δ 6.4 (m, 3H), δ 6.3 (m, 2H), δ 3.3 (m, 2H), δ 3.1 (m, 5H), δ 2.9 (m, 6H), δ 2.8 (m, 2H), δ 2.1 (m, 6H), δ 1.6 (m, 2H), δ 1.3 (m, 35H), δ 0.9 (m, 3H,); ^13^C NMR: δ 168.1, 166.0, 165.5, 136.3, 134.7, 131.3, 32.0, 29.8, 29.7, 29.4, 25.9, 22.7, 14.2.

PPD-3, pale yellow oil; ^1^H NMR: δ 6.8 (m, 1H), δ 6.7 (m, 1H), δ 6.3 (m, 5H), δ 3.5 (m, 1H), δ 3.3 (m, 3H), δ 3.0 (m, 3H), δ 2.8 (m, 2H), δ 2.1 (m, 1H), δ 1.6 (m, 4H), δ 1.3 (m, 83H), δ 0.8 (m, 9H); ^13^C NMR: δ 168.5, 166.0, 165.3, 136.6, 134.8, 131.0, 40.7, 37.9, 31.9, 29.7, 29.7, 29.6, 19.5, 29.4, 29.2, 28.8, 28.5, 26.8, 22.7, 14.1.

PPD-4, pale yellow oil; ^1^H NMR: δ 6.5 (m, 2H), δ 6.3 (m, 3H), δ 6.2 (m, 3H), δ 3.4 (m, 6H), δ 3.0 (m, 1H), δ 2.2 (m, 1H), δ 1.6 (m, 6H), δ 1.3 (m, 132H), δ 0.8 (m, 12H); ^13^C NMR: δ 136.9, 130.6, 40.7, 31.9, 29.7, 29.5, 29.4, 29.1, 28.8, 26.8, 22.7, 14.1.

PPD-5, yellow oil; ^1^H NMR: δ 6.9 (m, 5H), δ 6.7 (m, 1H), δ 6.4 (m, 4H), δ 6.3 (m, 12H), δ 3.5 (m, 1H), δ 3.4 (m, 12H), δ 3.0 (m, 7H), δ 2.8 (m, 2H), δ 2.1 (m, 4H), δ 1.6 (m, 14H), δ 1.3 (m, 229H), δ 0.9 (m, 20H); ^13^C NMR: δ 168.1, 166.0, 165.4, 136.5, 134.6, 134.0, 131.2, 40.7, 31.9, 29.7, 29.7, 29.6, 29.6, 29.5, 29.4, 29.2, 28.8, 26.8, 22.7, 14.1.

PPD-6, yellow oil; ^1^H NMR: δ 7.8 (m, 3H), δ 7.4 (m, 3H), δ 7.2 (m, 1H), δ 7.0 (m, 3H), δ 4.0 (m, 1H), δ 2.6(m, 1H),δ 2.4 (m, 2H), δ 2.2 (m, 9H), δ 1.6 (m, 1H), δ 1.3 (m, 16H), δ 0.9 (m, 2H); ^13^C NMR: δ 166.4, 137.8, 135.4, 133.5, 130.5, 130.0, 128.2, 127.9, 127.7, 127.6, 127.2, 126.9, 126.6, 126.1, 125.9, 125.7, 125.0, 124.1, 64.8, 32.0, 29.9, 29.8, 29.6, 29.5, 29.3, 28.7, 26.0, 22.8, 21.4, 19.4, 14.2.

PPD-7, yellow oil; ^1^H NMR: δ 8.0 (m, 7H), δ 7.8 (m, 26H), δ 7.5 (m, 29H), δ 7.3 (m, 24H), δ 7.0 (m, 8H), δ 6.4 (m, 7H), δ 6.1 (m, 1H), δ 5.8 (m, 1H), δ 4.1 (m, 5H), δ 3.9 (m, 15H), δ 2.8 (m, 6H), δ 2.7(m, 16H), δ 2.5 (m, 38H), δ 2.2 (m, 44H), δ 2.0 (m, 4H), δ 1.8 (m, 4H), δ 1.6 (m, 35H), δ 1.3 (m, 305H), δ 0.8 (m, 29H); ^13^C NMR: δ 130.5, 128.6, 128.1, 127.9, 127.7, 127.6, 127.2, 126.8, 126.5, 125.8, 125.7, 125.5, 124.9, 64.7, 31.9, 29.8, 29.7, 29.6, 29.5, 29.4, 29.3, 28.6, 25.9, 22.7, 21.7, 19.4, 14.2.

PPD-8, yellow oil; ^1^H NMR: δ 8.0 (m, 15H), δ 8.0 (m, 15H), δ 7.8 (m, 27H), δ 7.7 (m, 54H), δ 7.3 (m, 155H), δ 6.9 (m, 42H), δ 6.4 (m, 9H), δ 6.1 (m, 9H), δ 5.8 (m, 9H), δ 4.1 (m, 22H), δ 4.0 (m, 41H), δ 2.8 (m, 10H), δ 2.6 (m, 65H), δ 2.4 (m, 103H), δ 2.2 (m, 148H), δ 1.6 (m, 102H), δ 1.2 (m, 1062H), δ 0.9 (m, 109H); ^13^C NMR: δ 130.5, 128.9, 128.6, 128.5, 128.1, 128.9, 127.9, 127.7, 127.6, 127.2, 126.8, 126.5, 126.4, 125.8, 125.7, 125.5, 124.9, 124.1, 64.8, 32.0, 29.8, 29.7, 29.6, 29.5, 29.4, 29.4, 29.3, 28.6, 25.9, 22.7, 21.7, 20.6, 19.4, 14.2.

### 3.2. PPD Performance Evaluation

#### 3.2.1. Effect of PPD Addition on Pour Point of Crude Oil

**Figure 3:**
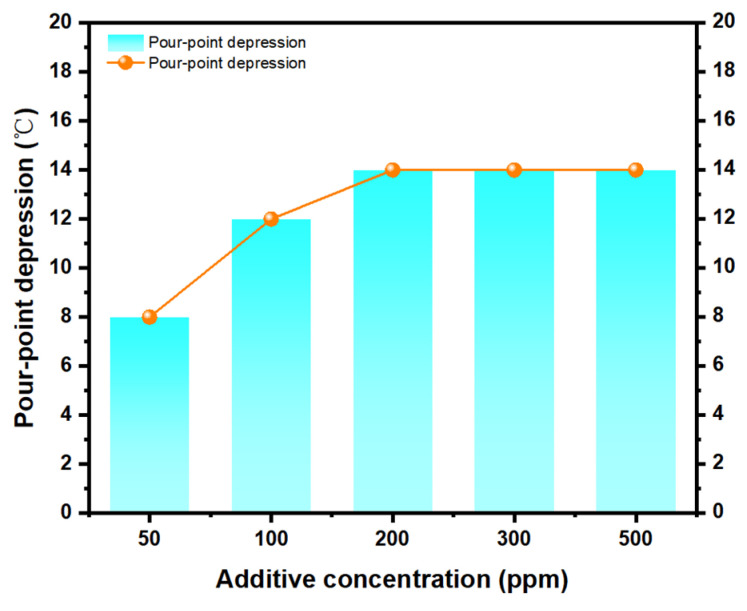
Pour points of shengli crude oil treated with PPD-8.

During the experiment, we investigated the effects of different types and concentrations of pour point depressants on the flowability of waxy crude oil. We placed the oil sample under 60 °C for 3 h and then allowed it to cool down steadily. We measured the effects of the eight pour point depressants on the pour point of waxy crude oil before and after addition at a concentration of 100 ppm. The results are shown in Table 4, indicating that PPD-8 had the best pour point depressant effect. Next, we measured the changes in the pour point of waxy crude oil with the addition of PPD-8 at concentrations of 50 ppm, 100 ppm, 200 ppm, 300 ppm, and 500 ppm, and the results are shown in Figure 3. It can be seen from the table that the best pour point depressant effect was achieved at a concentration of 200 ppm.

It can be distinctly observed that the pour point with pour point depressant was extremely lower than the pour point without additive PPD from Table 4, indicating that any type of pour point depressant displayed a great function on the high-pour-point crude oil. Compared with PPD-1~PPD-3, the polymer (PPD-4 and PPD-5) containing longer alkyl side chains can enhance the pour point ability by 7 °C and 8 °C, demonstrating that alkyl side chains can provide more approving adsorption sites for wax crystals. It has been shown that both PPD-7 and PPD-8 including aromatic units showed excellent performance in reducing the pour point by 9 °C and 12 °C. This can be best illustrated by the benzene ring can raise the solubility of the PPD and enhance the interactions between the PPD and paraffin in crude oil. It is not difficult to draw the conclusion that the selected PPD-8 at the concentration of 100 ppm possessed the most optimum property among the eight types of pour point depressant.

In Figure 3, it can be seen that the pour point depressant effect was poor when the concentration of the additive was low. When the concentration was increased to 200 ppm, the pour point depressant effect was better and the reduction in pour point reached 14 °C. If the concentration was further increased, the effect of the pour point depressant will not change much, which is not economically feasible and will increase the cost. Therefore, the optimal concentration for PPD-8 was 200 ppm.

Based on the above results, it can be concluded that the pour point depressant (PPD) should have appropriate alkyl chains that are hydrophobic and interact with the waxy microcrystals. The hydrophilic groups were exposed on the outer surface of the polymeric chain and acted as repellents for other waxy microcrystals, thereby inhibiting coalescence and the subsequent formation of larger waxy particles that could deposit. The long alkyl chain of the PPD played an important role in its interaction with wax crystals, as it can act as the nucleation center for wax precipitation and increase the number of small wax crystals in the crude oil, thereby preventing the formation of large network structures. Additionally, the long alkyl chain can also form eutectic with wax, altering the crystal orientation and inhibiting further wax crystal growth. Polypropylene acid ester with a long alkyl chain belongs to a comb-shaped polymer and possesses excellent shear resistance. The elongated aliphatic chains continuously extend around the resin and asphaltene, lowering the polarity of the surrounding environment and acting as a shield, thus preventing the aggregation and accumulation of resin and asphaltene fragments, resulting in a reduction in crude oil viscosity. In addition to the long alkyl chain structure, the polar functional groups in the PPD also have a significant impact on its efficiency. After the PPD is adsorbed onto the wax crystals, its polar functional groups can repel other wax crystals, preventing them from aggregating into larger clusters, thereby improving the dispersion of wax crystals and enhancing the flowability of crude oil. The polar functional groups in maleic anhydride and acrylic acid esters can effectively interact with the natural components of pour point depression of resin and asphaltene in crude oil. The PPD enters the resin and asphaltene fragments through intermolecular forces, resulting in stronger hydrogen bonding. In the meanwhile, the oxygen-containing polar functional groups also improve the PPD’s resistance to repeated heating and shear stress. Moreover, the benzene ring of chains can interact with asphaltenes through a π–π stacking attractive force. The combined action of the alkane carbon chain, polar functional groups, and benzene ring prevent the mutual binding of wax crystals, thus reducing the pour point, viscosity, and yield stress of the crude oil. Therefore, the low-temperature flowability of the crude oil is significantly improved.

#### 3.2.2. Wax Crystal Pattern of Crude Oil after Adding PPD-8

**Figure 4:**
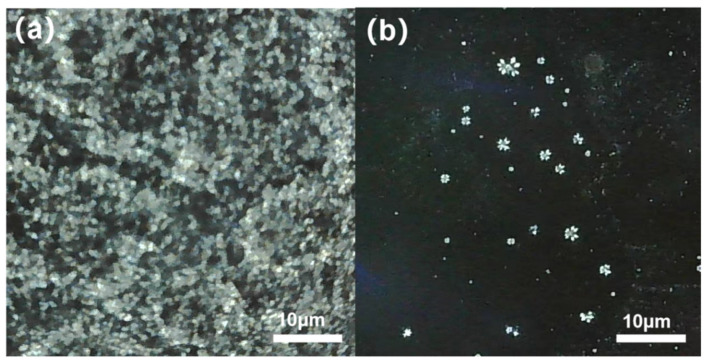
Polarized microscopy images of crude oil without and with PPD-8. (**a**) virgin crude oil (**b**) crude oil with 200 ppm concentration of pour point depressant at 20 °C.

Figure 4 shows the polarized microscopy images of the crude oil without pour point depressant and with 200 ppm of pour point depressant at 20 °C. From the figure, it can be seen that the wax crystal size in the crude oil without pour point depressant was small, the wax crystals were relatively loose, and they were distributed more evenly in the oil phase. After adding PPD-8 pour point depressant, the size of the wax crystal particles in the crude oil became larger, with agglomeration forming large spherical crystal aggregates. The addition of pour point depressant changed the crystallization mode of wax in the crude oil, decreased the amount of wrapped liquid oil, weakened the flocculation ability of wax crystal aggregates, reduced the strength of the spatial grid structure, and increased the flowability of crude oil.

### 3.3. Analysis of the PPD Mechanism

The long carbon chain structure in PPD easily formed eutectic with wax crystals in crude oil. The polar groups in acrylate compounds enhanced the intermolecular repulsion among the wax crystals, while the introduction of benzene rings increased the solubility of wax crystals, preventing them from agglomerating into wax deposits and enhancing the anti-deposition capability. The addition of PPD changed the crystallization pattern of the crude oil, causing the wax crystals to tend to aggregate into clusters, lowering the surface energy and reducing the spatial structural strength. This improvement at a macroscopic level manifested as an enhancement in the low-temperature flowability of the crude oil.

## 4. Conclusions

This study demonstrated that modified maleic anhydride co-polymers can be available pour point depressants for waxy crude oil. In this study, eight different types of pour point depressants were synthesized by copolymerizing maleic anhydride with different monomers and characterized using techniques such as FT-IR and NMR. The results showed that the addition of pour point depressants successfully reduced the pour point of the waxy crude oil, with an optimal concentration of 200 ppm for some of the tested compounds. The addition of pour point depressants changed the crystallization mode of wax in the crude oil, resulting in larger and more densely packed wax crystal aggregates, which improved the flowability of the crude oil. The study also found that the performance of the pour point depressants can be optimized by changing the hydrophobic parts and hydrophilic part of the polymer structure. Therefore, modified maleic anhydride co-polymers have the potential to be used as effective pour point depressants for waxy crude oil.

## Figures and Tables

**Table 1:** Physical characteristics of shengli crude oil.

Properties	Shengli Crude Oil
Density at 20 °C (g/cm^3^)	0.85
Pour point (°C)	32
Wax content (wt%)	21.23
Resin content (wt%)	2.17
Asphaltene content (wt%)	0.89
IBP (°C)	60.5

**Table 2:** The selections of monomers.

Monomer A	Monomer B	Monomer C	Products
Acrylic acid	Propyl acrylate	None	PPD-1
Acrylic acid	Propyl acrylate	Maleic anhydride	PPD-2
Methyl methacrylate	Propyl acrylate	Maleic anhydride	PPD-3
Acrylic acid	Octadecyl acrylate	Maleic anhydride	PPD-4
Methyl acrylate	Nonadecyl acrylate	Maleic anhydride	PPD-5
Methyl methacrylate	Propyl acrylate	Diethyl succinate	PPD-6
Methyl methacrylate	Propyl acrylate	Dibenzyl maleate	PPD-7
Methyl acrylate	Nonadecyl acrylate	EsterDibenzyl	PPD-8

**Table 3:** Molecular weights and yields of pour point depressants.

Additive	M_w_ (g/mol)	Yield (%)
PPD-1	34,958	90
PPD-2	38,458	88
PPD-3	41,520	89
PPD-4	41,330	91
PPD-5	42,339	87
PPD-6	39,415	81
PPD-7	38,965	82
PPD-8	40,134	85

**Table 4:** Effect of pour point depressants at a concentration of 100 ppm on Shengli crude oil (PP = 32 °C).

Additive	Pour Point with PPD/°C	Pour-Point Depression/°C
PPD-1	26	6
PPD-2	28	4
PPD-3	27	5
PPD-4	25	7
PPD-5	24	8
PPD-6	26	6
PPD-7	23	9
PPD-8	20	12

## Data Availability

The data used to support the findings of this study are available from the corresponding author upon request.

## Supplementary Materials

The following supporting information can be downloaded at: https://www.mdpi.com/article/10.3390/polym15193898/s1. Ref. [37] is cited in the supplementary materials.
