# Supplementary material for: Synthesis and Performance Testing of Maleic Anhydride–Ene Monomers Multicomponent Co-Polymers as Pour Point Depressant for Crude Oil
Source: Polymers (Basel). 2023 Sep 27;15(19):3898. doi: 10.3390/polym15193898 (PMC10574900; doi:10.3390/polym15193898)
Supplement: Supplementary file 1 [file polymers-15-03898-s001.zip › polymers-2593461-supplementary.pdf]

Instrumentation

The Fourier transform infrared spectrometry (FT-IR) analyses were measured on a Nicolet 5700 spectrophotometer in the range 400-4000 cm^{-1}. ^{1}H NMR and ^{13}C NMR spectra were recorded on 400 MHz (Bruker) instrument, $CDCl_3$ (7.26 ppm for ^{1}H NMR, 77.16 ppm for ^{13}C NMR) was used as a reference [1,2,3].

1. **NMR spectra of products**

Pour Point Depressant 1（PPD-1）

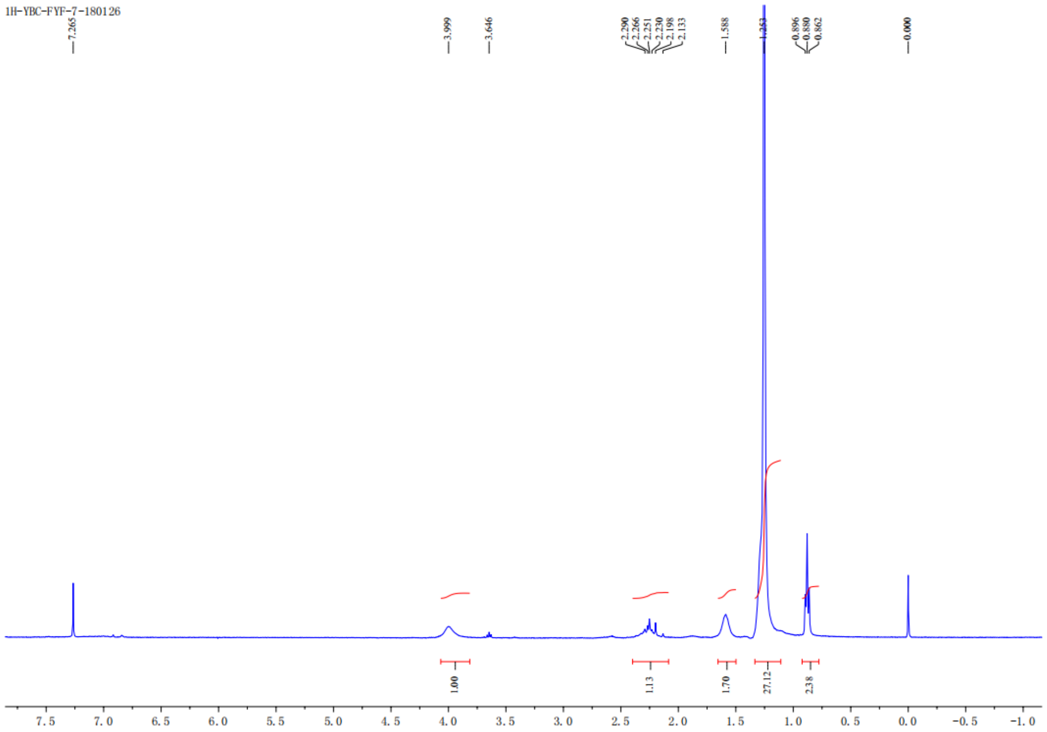

Figure S1. ^{1}H NMR spectra of product PPD-1

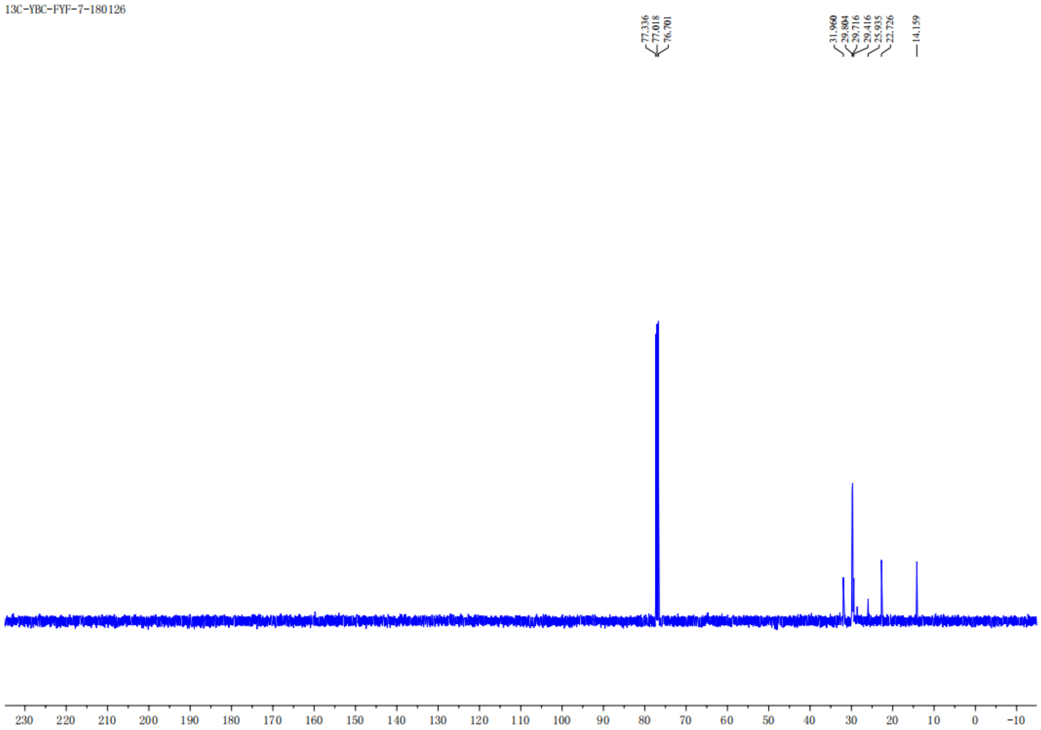

Figure S2. ^{13}C NMR spectra of product PPD-1

Pour Point Depressant 2（PPD-2）

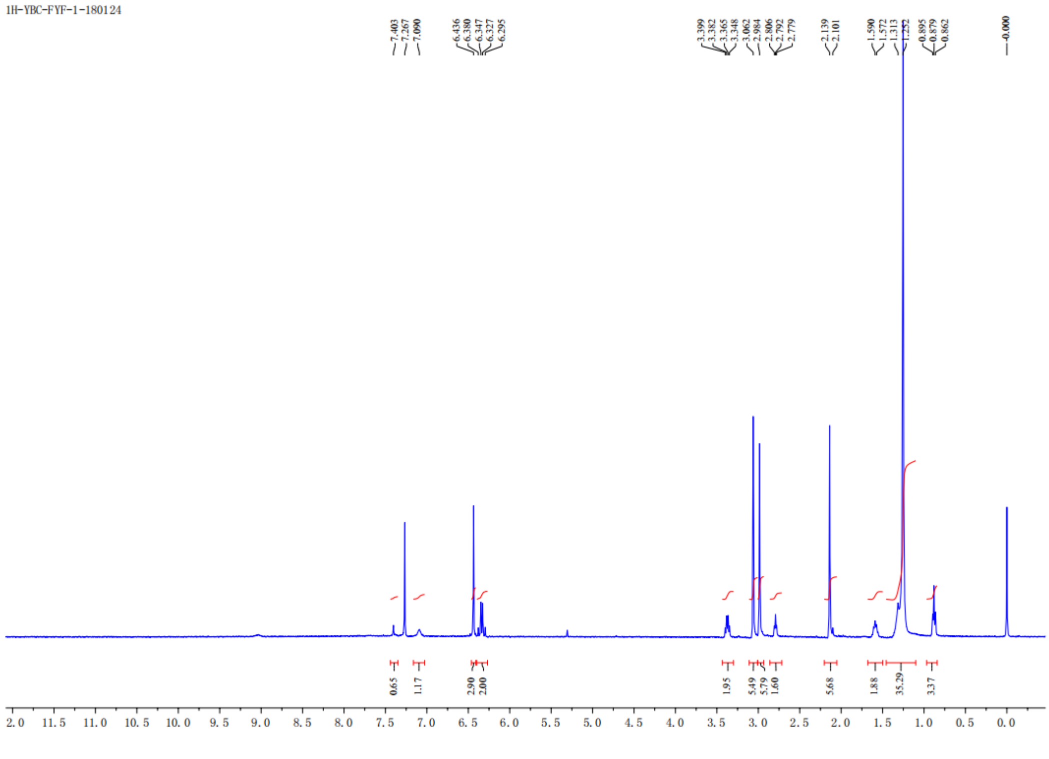

Figure S3. ^{1}H NMR spectra of product PPD-2

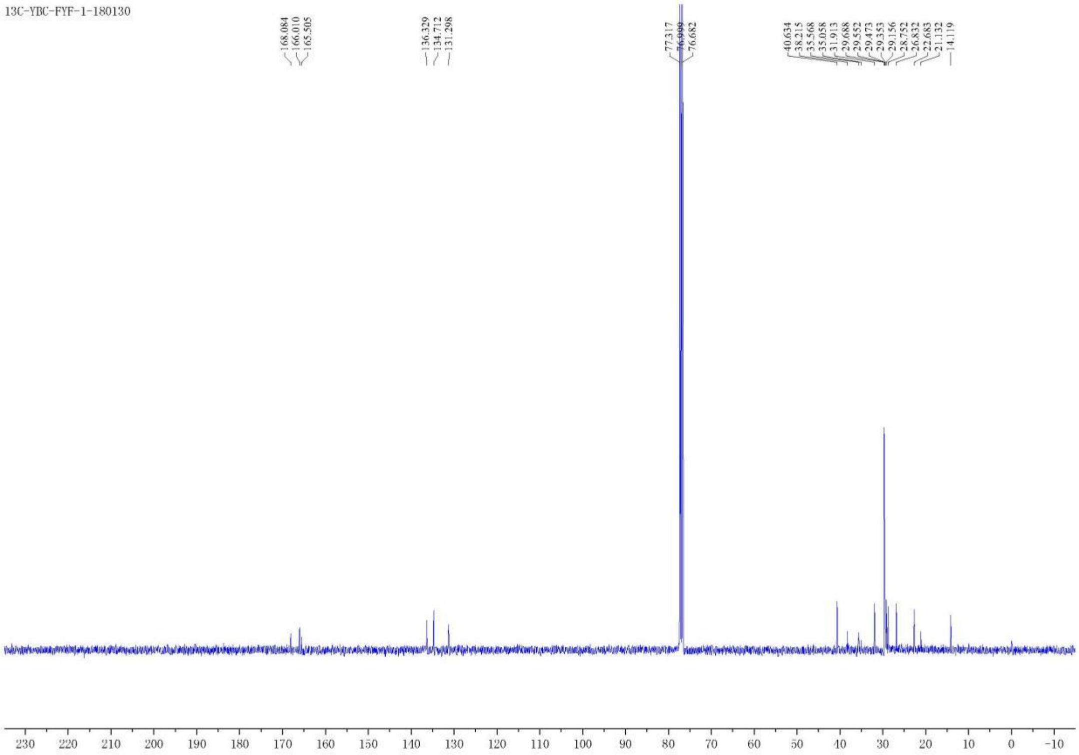

Figure S4. ^{13}C NMR spectra of product PPD-2

Pour Point Depressant 3（PPD-3）

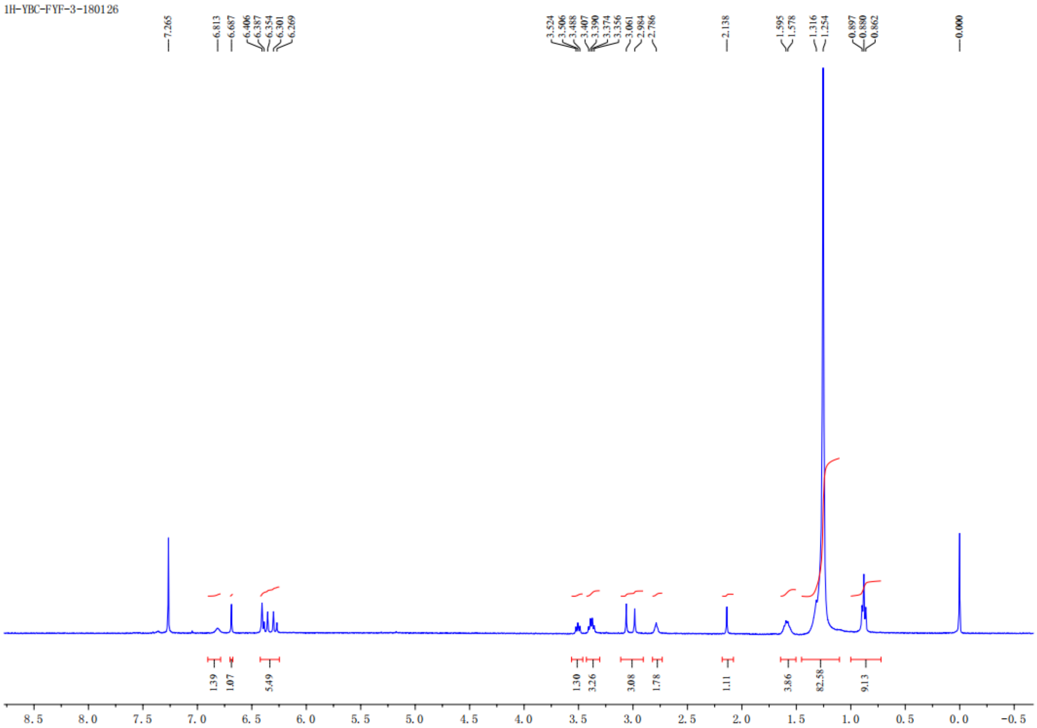

Figure S5. ^{1}H NMR spectra of product PPD-3

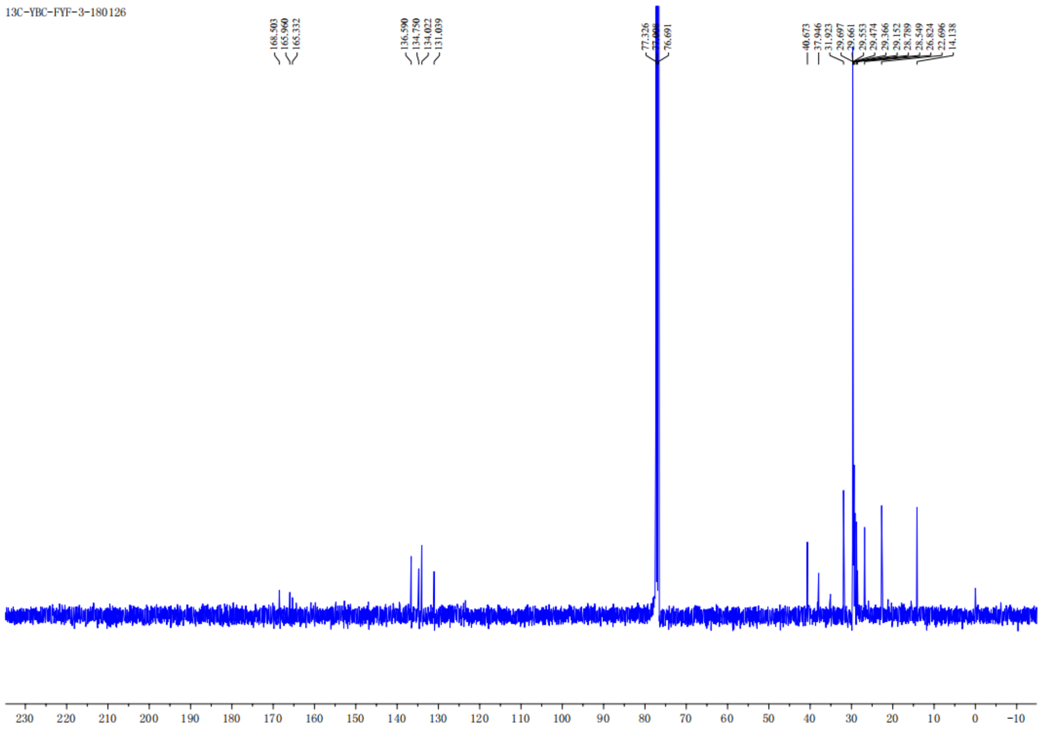

Figure S6. ^{13}C NMR spectra of product PPD-3

Pour Point Depressant 4（PPD-4）

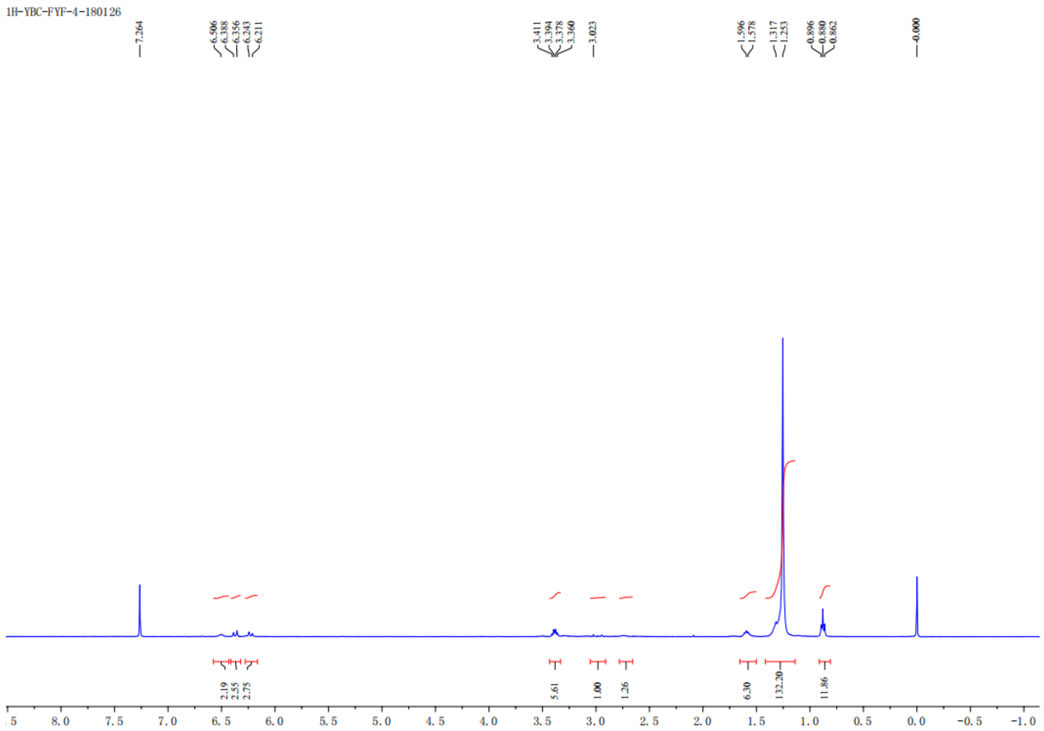

Figure S7. ^{1}H NMR spectra of product PPD-4

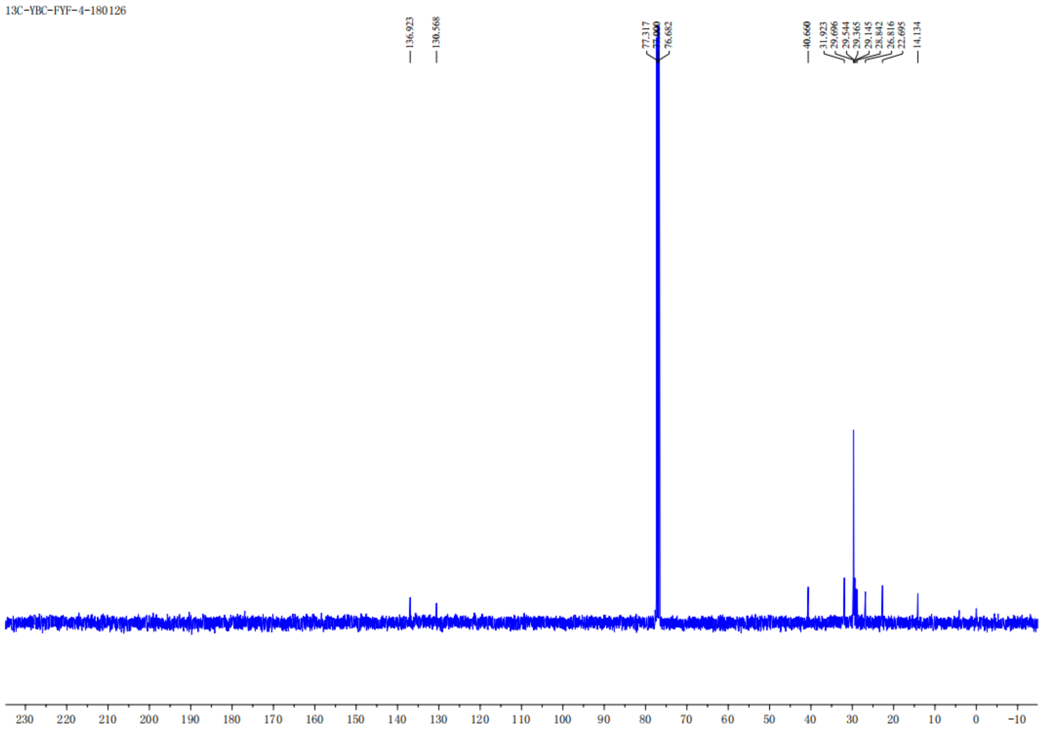

Figure S8 ^{13}C NMR spectra of product PPD-4

Pour Point Depressant 5（PPD-5）

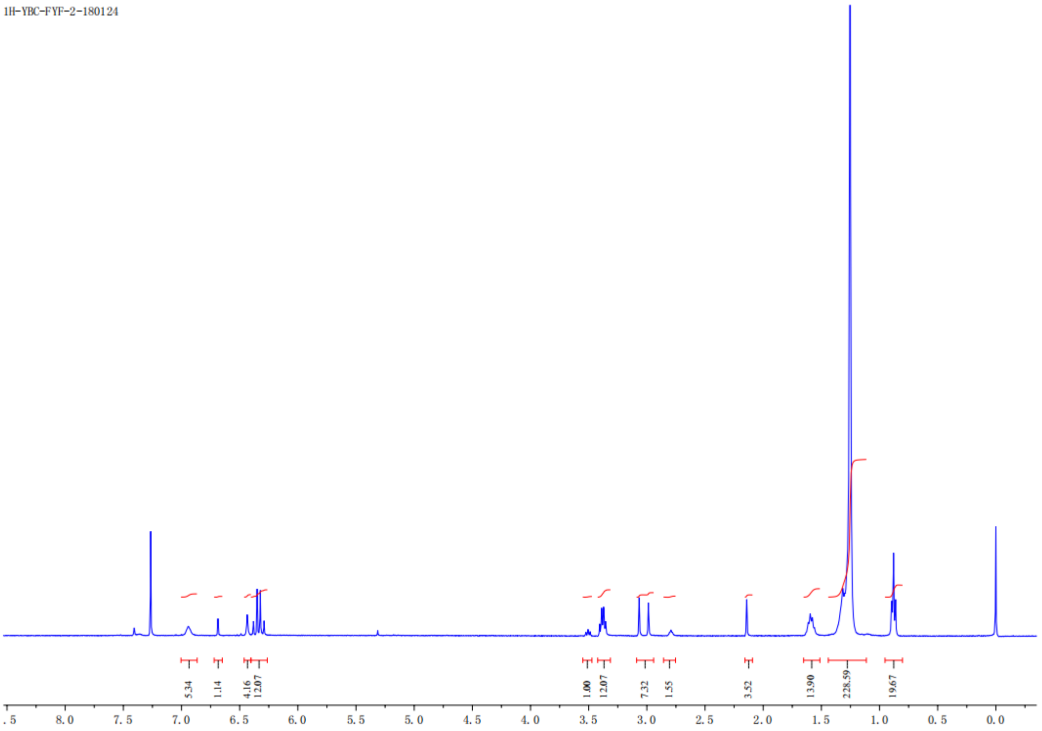

Figure S9. ^{1}H NMR spectra of product PPD-5

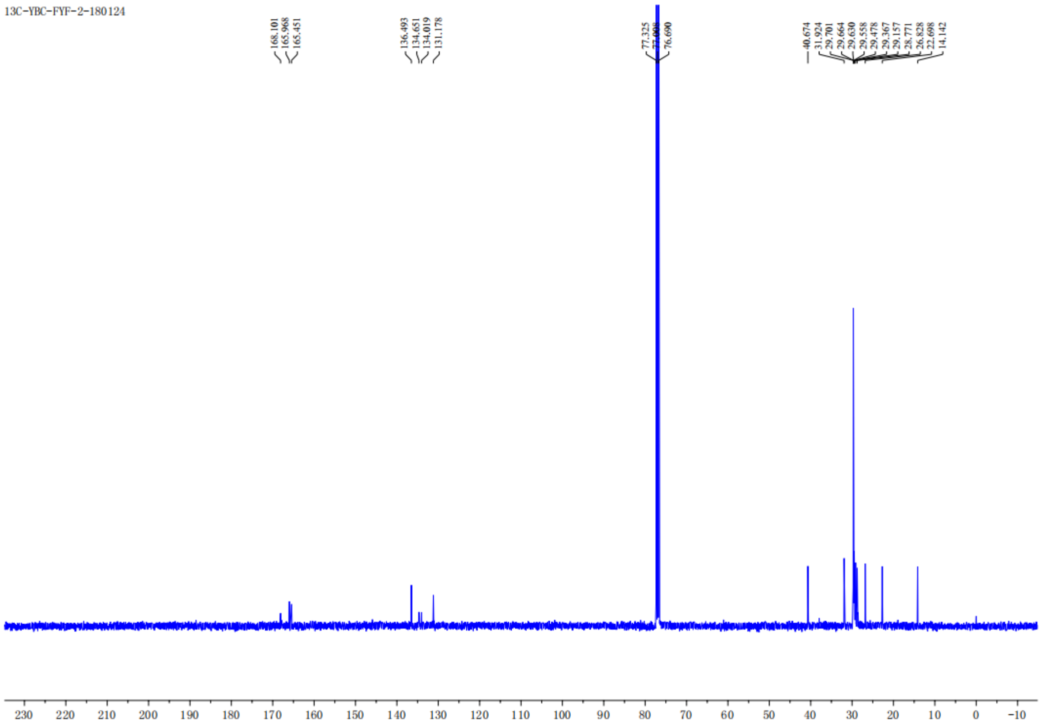

Figure S10. ^{13}C NMR spectra of product PPD-5

Pour Point Depressant 6（PPD-6）

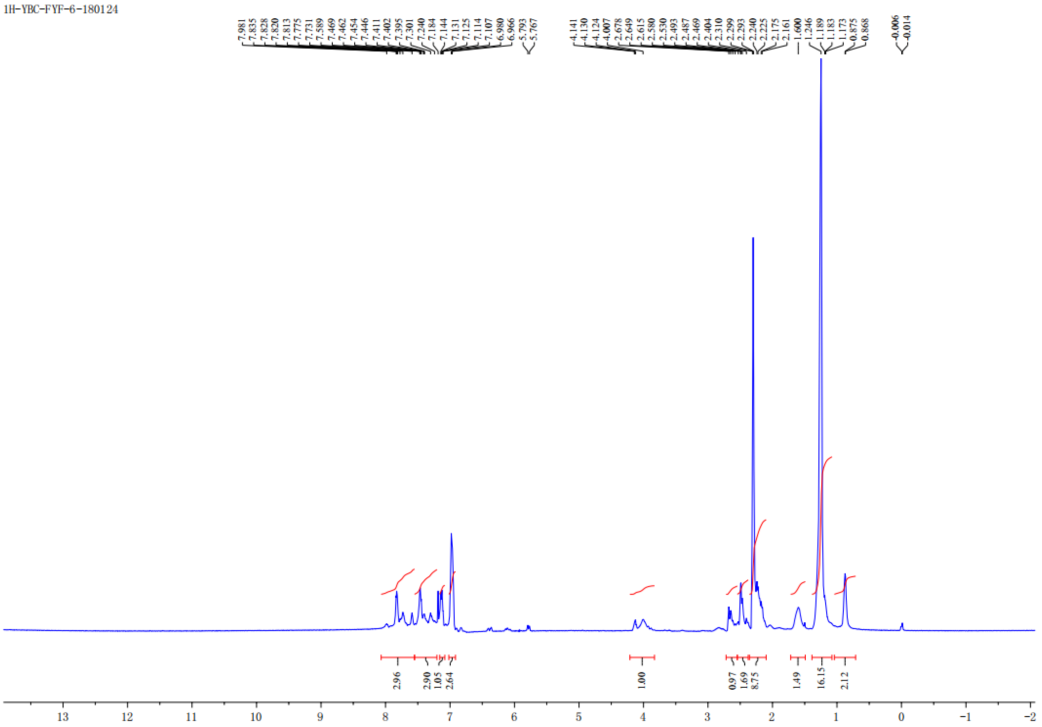

Figure S11. ^{1}H NMR spectra of product PPD-6

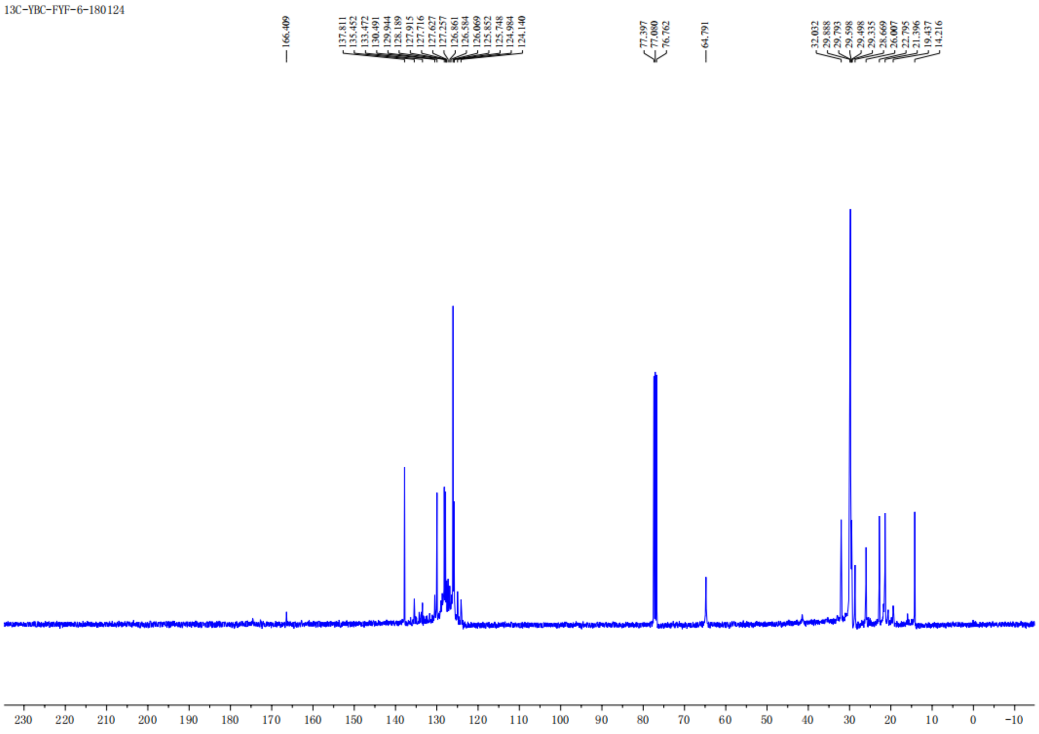

Figure S12. ^{13}C NMR spectra of product PPD-6

Pour Point Depressant 7（PPD-7）

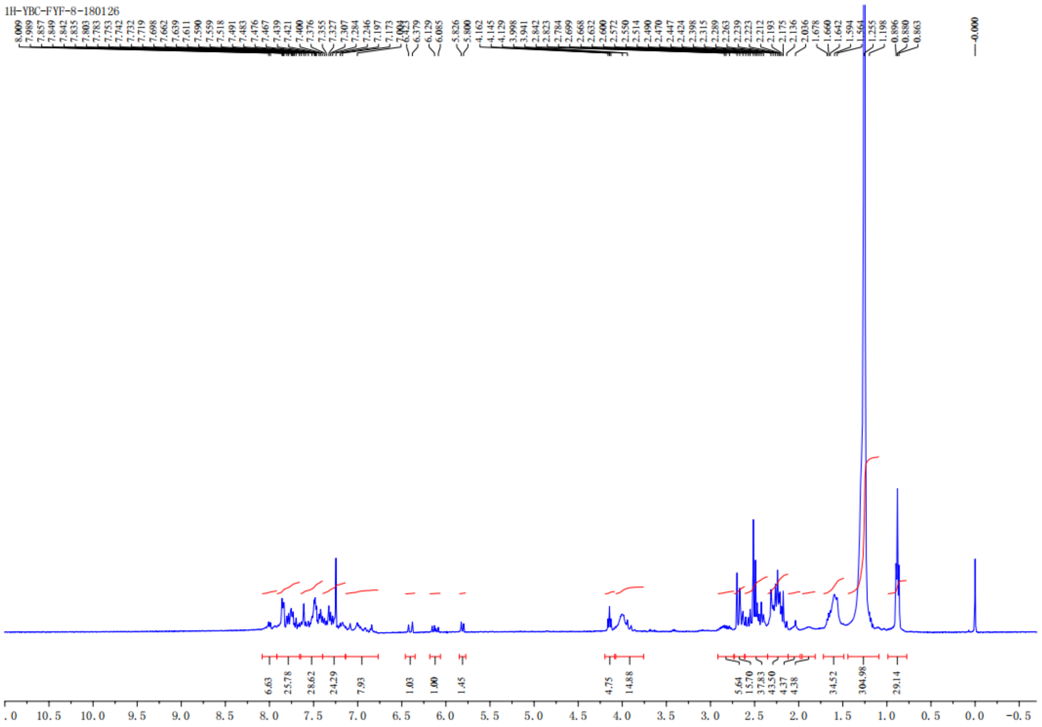

Figure S13. ^{1}H NMR spectra of product PPD-7

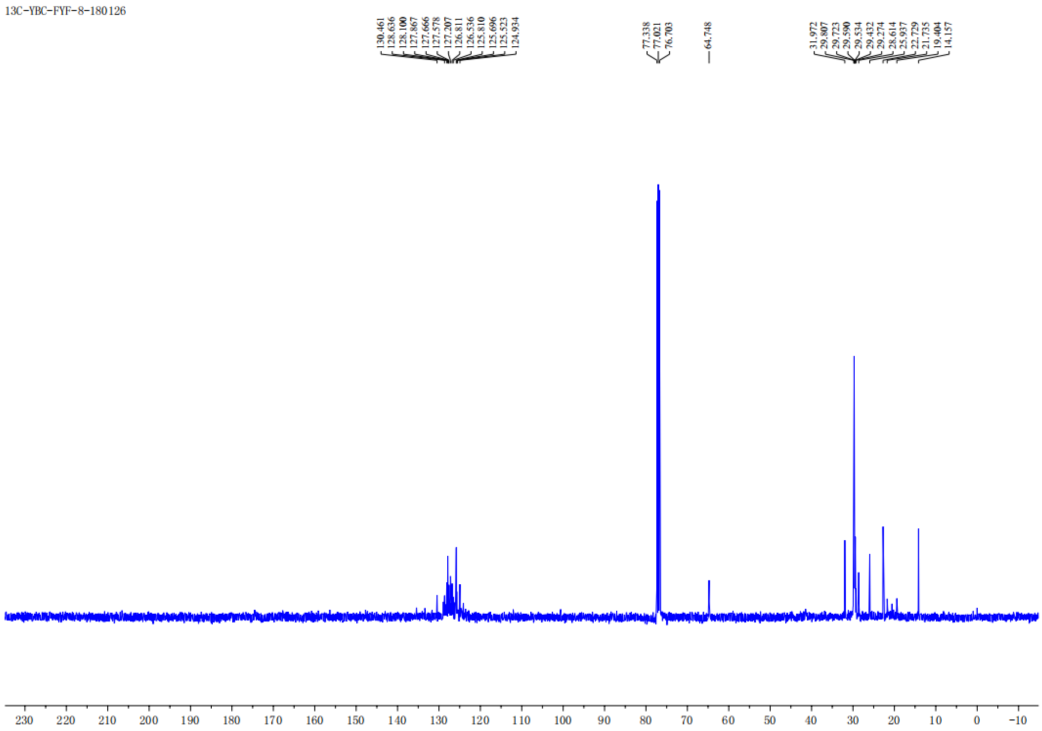

Figure S14. ^{13}C NMR spectra of product PPD-7

Pour Point Depressant 8（PPD-8）

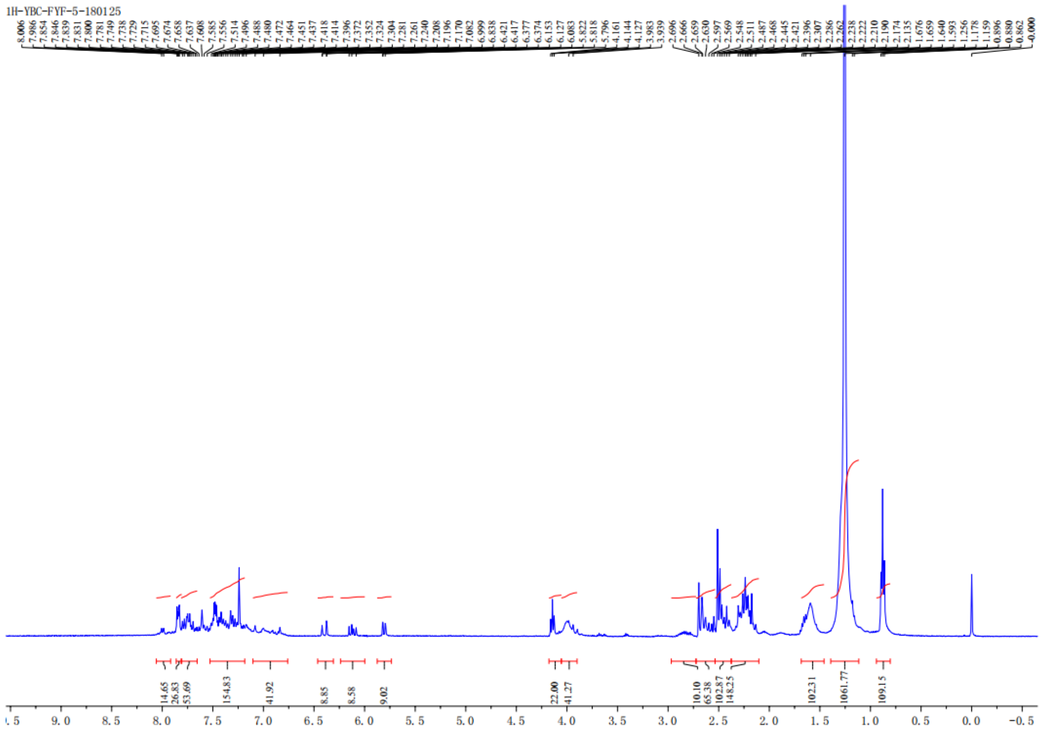

Figure S15. ^{1}H NMR spectra of product PPD-8

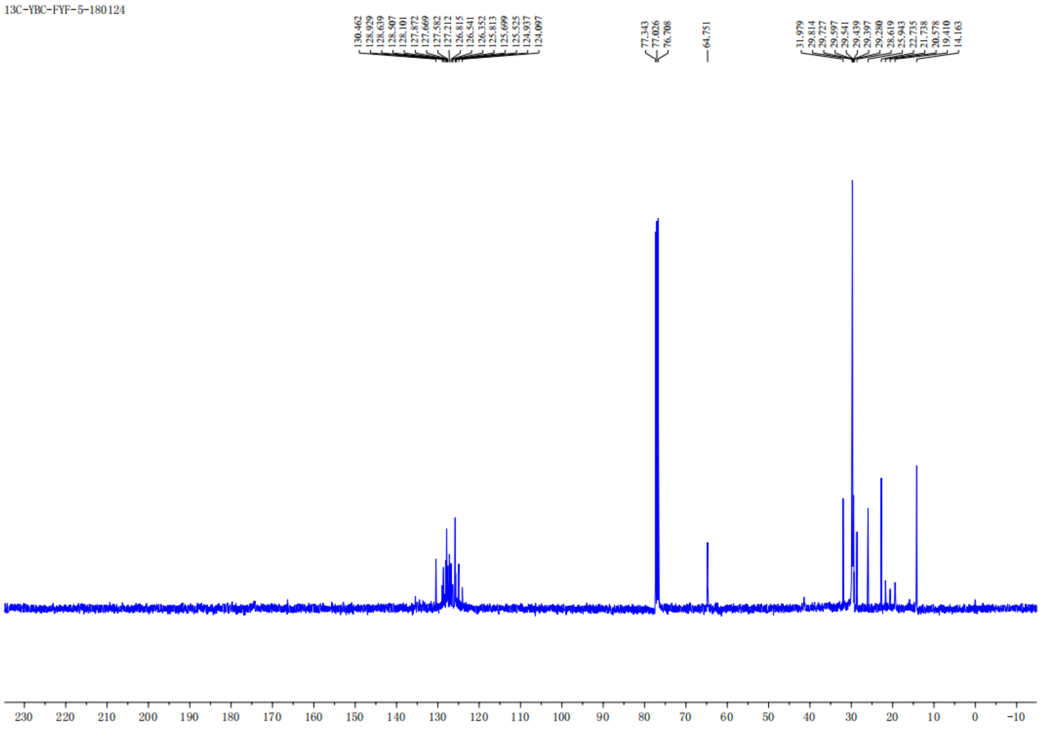

Figure S16. ^{13}C NMR spectra of product PPD-8

2. **FTIR spectra of products**

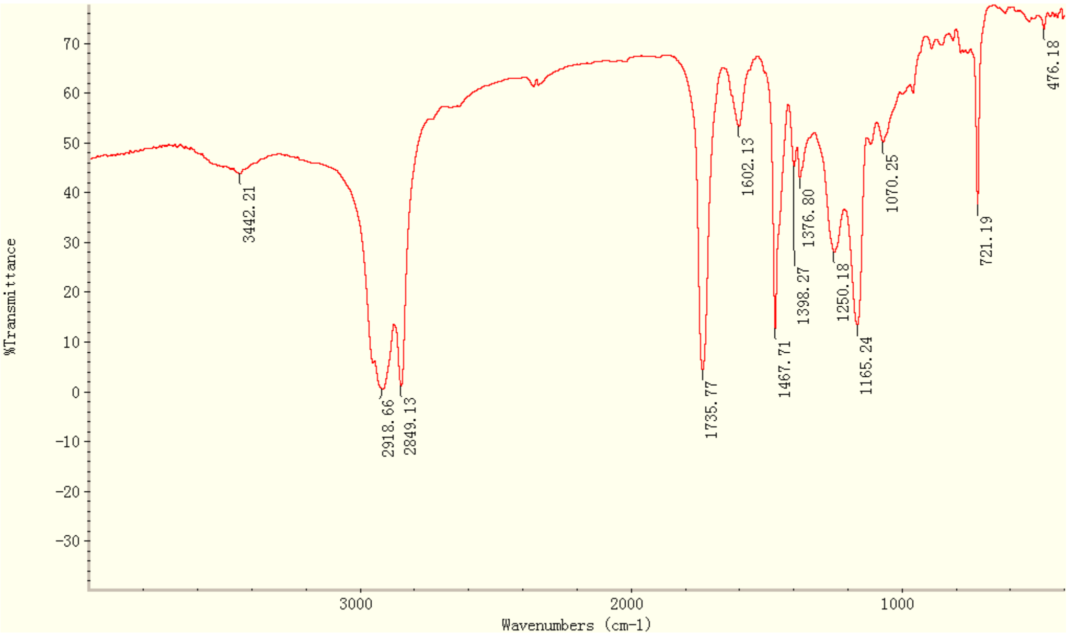

Figure S17. FTIR spectra of PPD-1

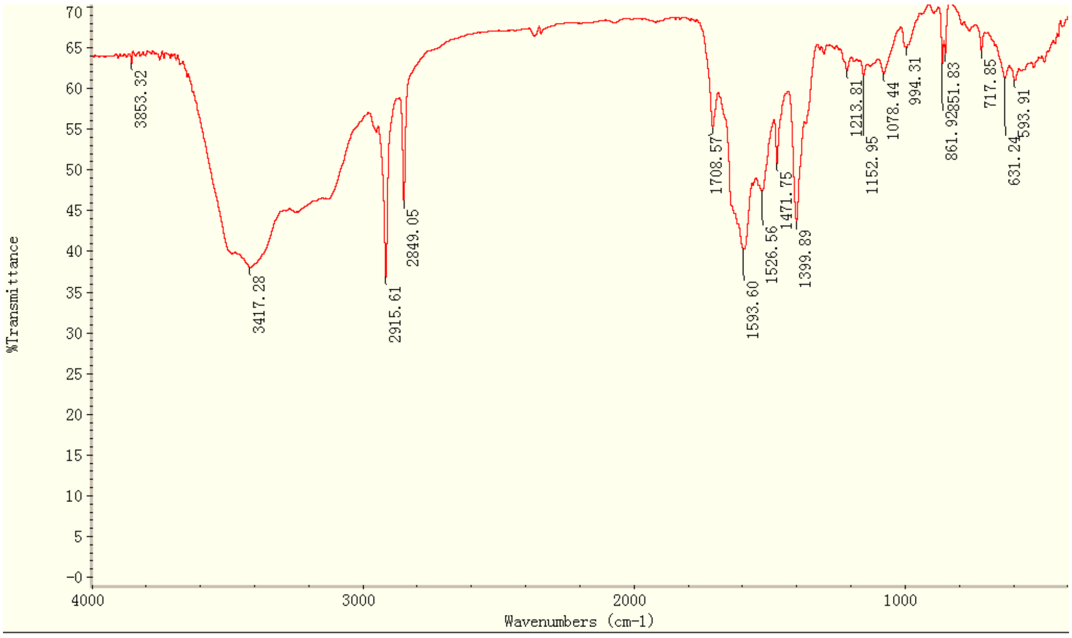

Figure S18. FTIR spectra of PPD-2

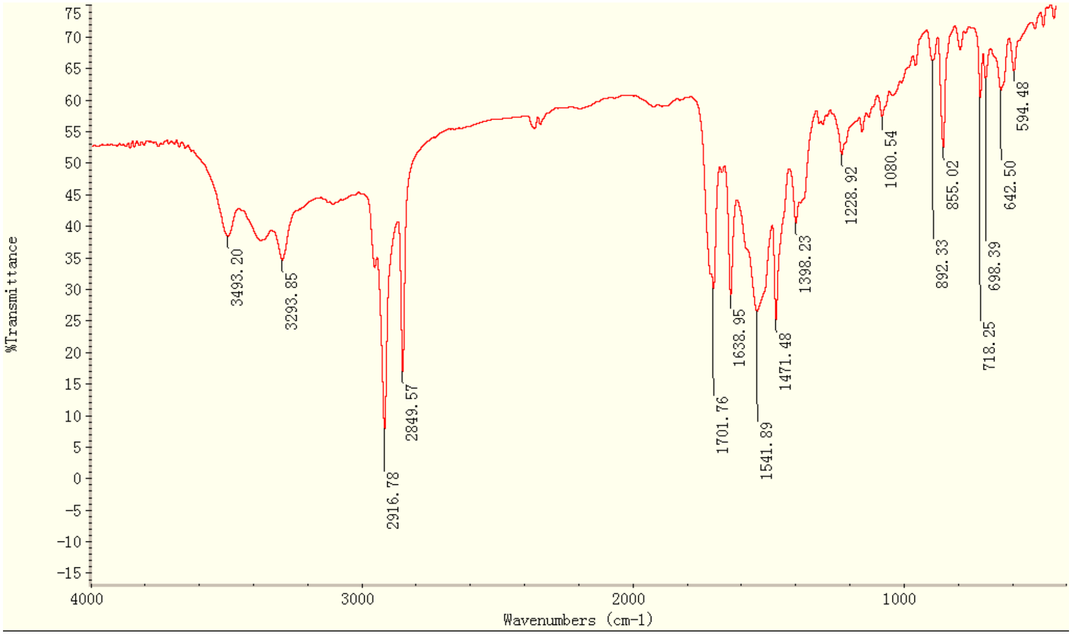

Figure S19. FTIR spectra of PPD-3

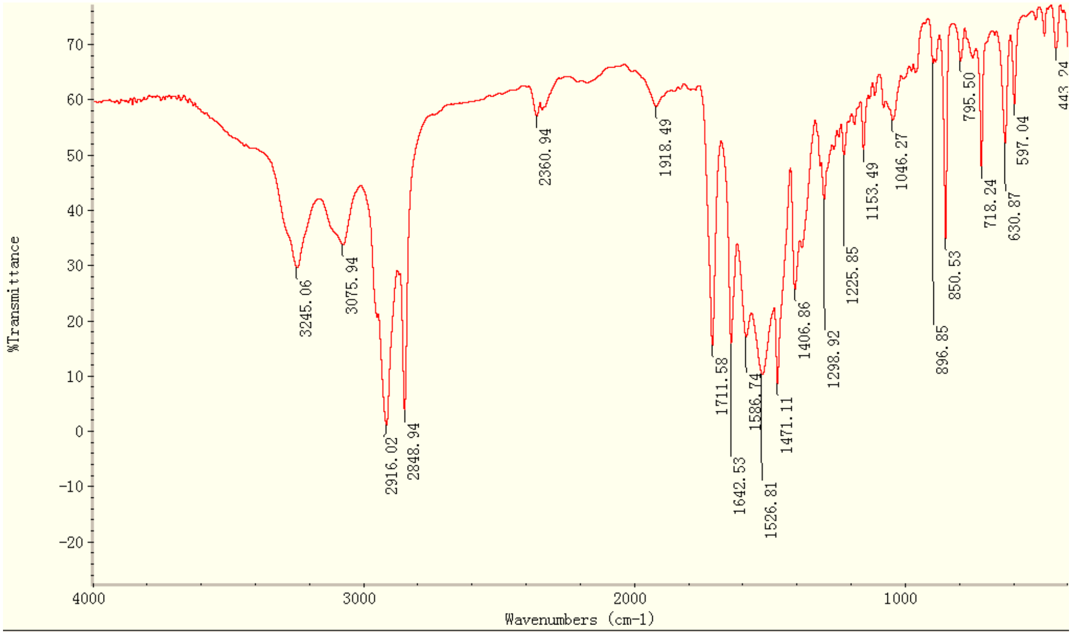

Figure S20. FTIR spectra of PPD-4

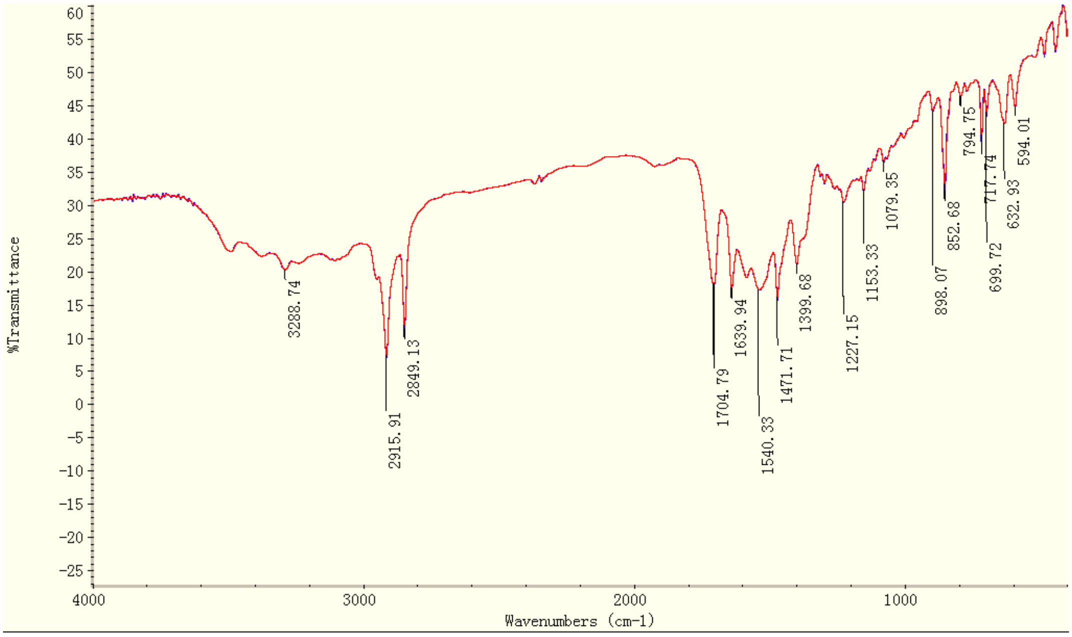

Figure S21. FTIR spectra of PPD-5

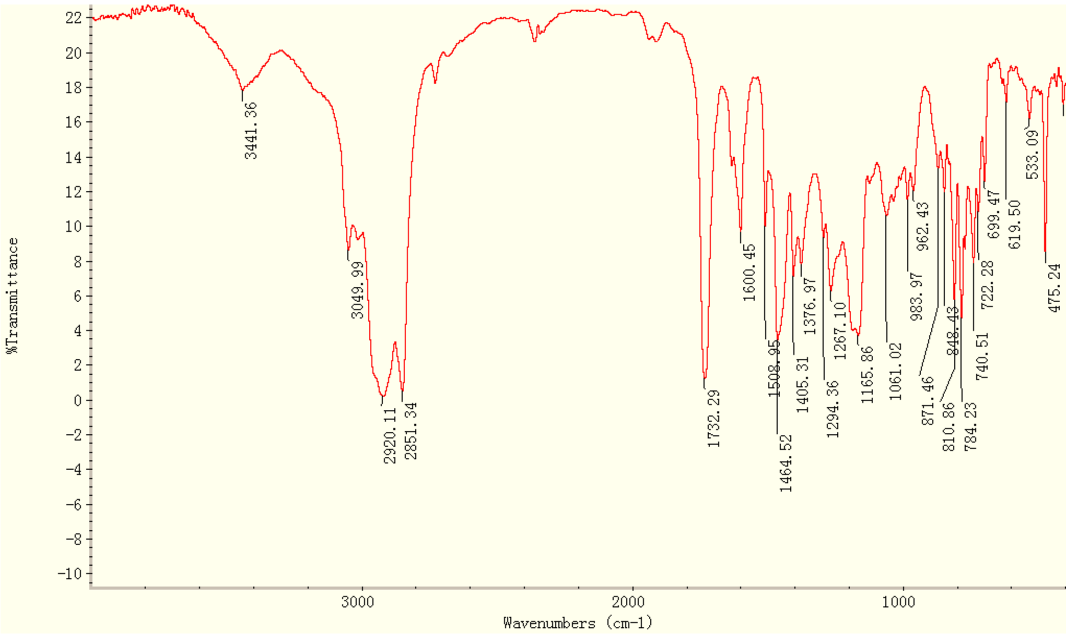

Figure S22. FTIR spectra of PPD-6

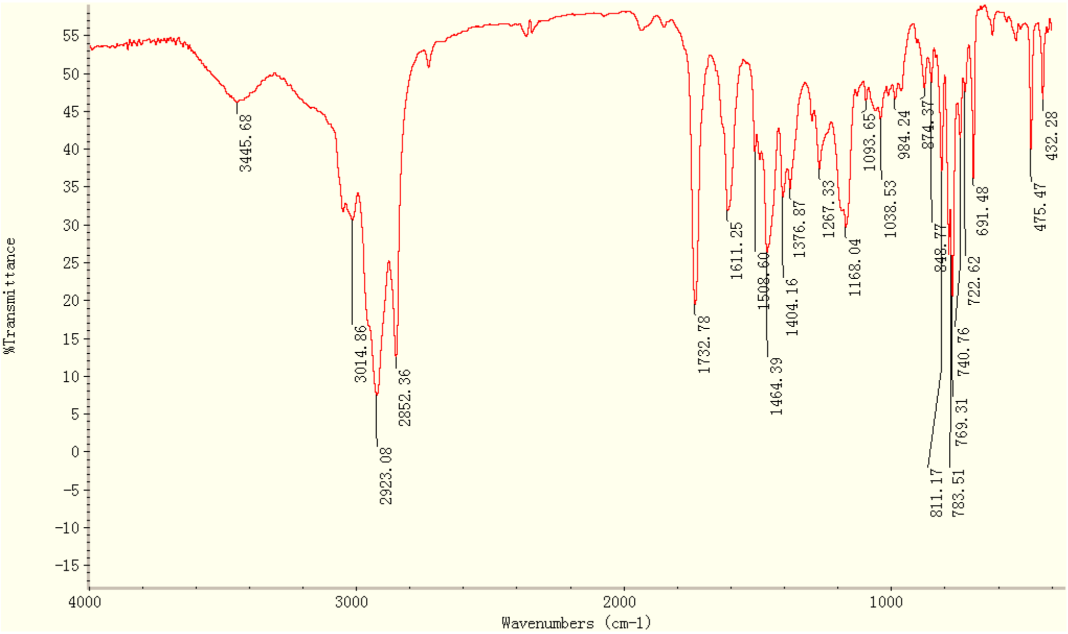

Figure S23. FTIR spectra of PPD-7

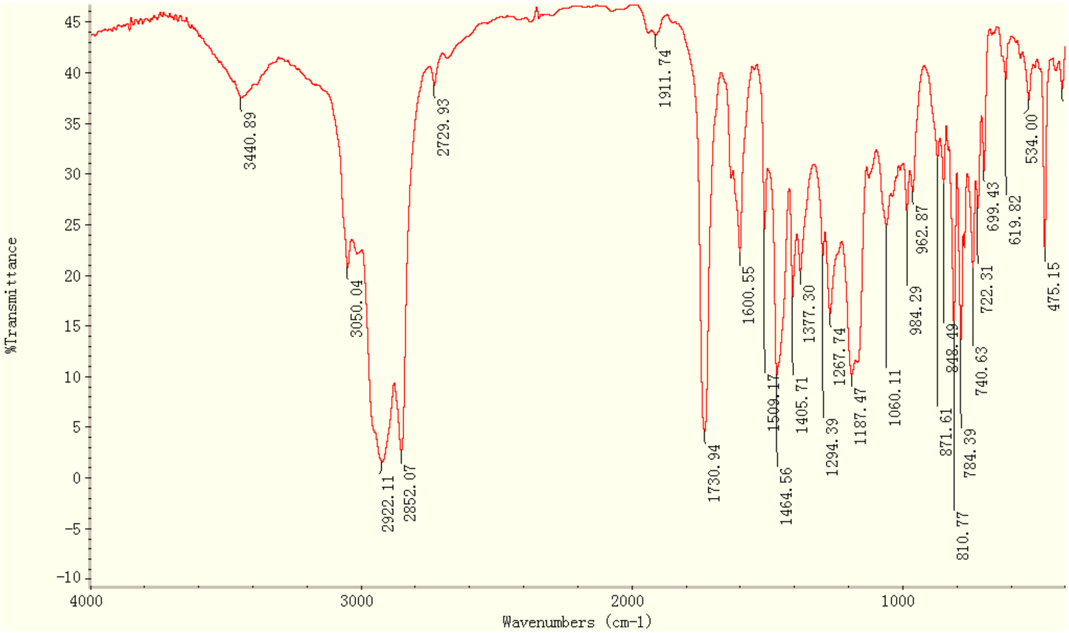

Figure S24. FTIR spectra of PPD-8

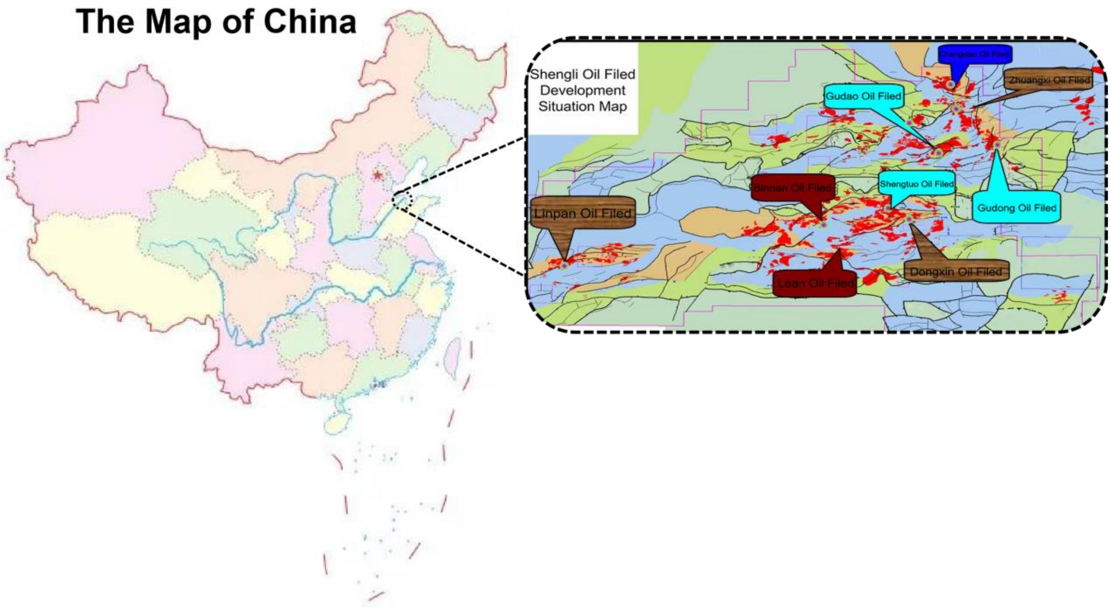

Figure S25. The map locating the Shengli oil field in China.

References

1. Xie, M.; Chen, F.; Liu. Synthesis and evaluation of benzyl methacrylate-methacrylate copolymers as pour point depressant in diesel fuel. *Fuel.* **2019**, *255*, 115880.
2. Fang, L.; Zhang, X.; Ma, J. Investigation into a pour point depressant for Shengli crude oil. *Industrial & engineering chemistry research.* **2012**, *51(36)*, 11605-11612.
3. Pucko, I.; Racar, M.; Faraguna, F. Synthesis, characterization, and performance of alkyl methacrylates and tert-butylaminoethyl methacrylate tetra polymers as pour point depressants for diesel Influence of polymer composition and molecular weight. *Fuel*. **2022**, *324*, 124821.
